# The impact of urbanisation on community structure, gene abundance and transcription rates of microbes in upland swamps of Eastern Australia

**DOI:** 10.1371/journal.pone.0213275

**Published:** 2019-03-04

**Authors:** Nicole A. Christiansen, Kirstie A. Fryirs, Timothy J. Green, Grant C. Hose

**Affiliations:** 1 Department of Biological Sciences, Macquarie University, North Ryde, NSW, Australia; 2 Department of Environmental Sciences, Macquarie University, North Ryde, NSW, Australia; Assam University, INDIA

## Abstract

The Temperate Highland Peat Swamps on Sandstone of the Sydney Basin occur in the headwaters of Sydney’s drinking water catchments and are listed as endangered ecosystems, yet they have suffered habitat losses and degradation due to human impacts such as urbanisation. Despite ongoing efforts to restore and better protect upland swamps, they remain poorly understood, potentially hindering the effectiveness of management efforts. Essential to overall ecosystem function and the provision of services for human and environmental benefit are the microbial component of wetland ecosystems. In the case of these swamps, the microbes, have not yet been studied. Here, we investigated differences in the microbial community of upland swamps in urbanised catchments compared to swamps from natural catchments in the Blue Mountains. A total of twelve swamps were sampled, six from within urbanised catchments and six with intact vegetation catchments, to compare sediment conditions and microbial community and genes expression and abundances. Catchment impervious area and number of stormwater drains entering a swamp, indicators for urbanisation, positively correlated with the pH and ammonium concentration of swamp sediment. Community analysis of the 16S rRNA gene (T-RFLP, qPCR) revealed the elevated pH of urbanised swamps coincided with changes to the abundance of bacteria and archaea. Furthermore, RT-qPCR revealed genes involved in carbon cycling (*mcrA* & *pmoA*) were more likely to be found in urbanised swamps. Taken together, our results indicate that urbanisation of the Blue Mountains is impacting the environmental services provided by the microbial community of upland swamps in the Sydney Basin.

## Introduction

Wetlands, including swamps, bogs and fens, are important landscape features that serve to regulate water flow, improve water quality, provide wildlife habitat and store carbon [1]. They can buffer the effects of activities in a catchment on downstream aquatic ecosystems and, consequently, are often prioritised for restoration and conservation [2, 3].

Wetlands, classified as Temperate Highland Peat Swamps on Sandstone (THPSS), are a common feature of the low relief plateaus that surround the Sydney geological Basin, in South East Australia [4]. The THPSS, hereafter called swamps, are ecologically important; they occur only within restricted ranges, harbour endangered species and unique plant communities [5] and provide ecosystem services such as carbon storage and regulation of water quantity and quality [6, 7]. A defining feature of these systems is the peat-like sediments that arise from the accumulation of organic matter and slow microbial degradation in the anoxic, water logged soils.

Despite their inherent values, many swamps have been destroyed or degraded by human activities [8] leading to the swamps being listed as a threatened ecological community under state and federal legislations [9, 10]. Swamps located within the developed areas of the Blue Mountains approximately 100 km west of Sydney NSW, have been particularly degraded due to urbanisation. Factors associated with urbanisation, such as increased impervious area in the catchment and construction of stormwater drains and bores, have altered surface water chemistry [11], water table depths, caused erosion and channelisation, and altered the sediment profiles of the swamps in the Blue Mountains [12].

Restoration efforts have attempted to re-establish the hydrological processes within swamps by stopping or reversing erosion or buffering flashy stormwater flows [13, 14]. While restoration of swamp hydrology and sedimentary structure is critical to restoring ecological functions [12, 13, 15], other symptoms of urbanisation, such as altered water chemistry, must also be addressed. High concentrations of nutrients [16] are a common side effect of urbanisation in surface waters. Higher pH values in runoff from naturally acidic catchments, such as the study area, have also been reported[17, 18]. Nutrient concentration and pH levels have been shown to affect the microbial community structure in sediments generally [19]. In wetlands, pH is a key determinant of microbial community, greater than biogeography or wetland type [20]. High nutrient levels in wetlands increase the microbial mediated release of organic matter [21] and provide more favourable conditions for greenhouse gas production [22–24]. Since the microbial community is fundamental to carbon and nitrogen cycling, changes to these communities may be significant. Altered ecological function of these swamps may have implications for downstream water quality [1] and even shift wetlands from being carbon sinks to greenhouse gas sources [6, 25].

In other environments, urbanisation causes shifts in the microbial community resulting in altered carbon and nitrogen cycling [26–28]. Urban ecosystems (lawns) in arid and semiarid landscapes can have increased carbon cycling and altered carbon budgets and microbial biomass when compared to natural ecosystems and agricultural land uses [26]. Urbanised lake surface sediments showed microbial community differences mainly due to increased nitrogen loads [27]. Microbial communities from the urban areas of China’s Jailing River system were influenced by discharges from urban development with phosphorous, ammonia, iron and zinc concentrations being significant determinants of community structure [28].

The aim of this study is to compare the microbial communities and functions of upland swamps in intact and urbanised catchments. Our hypothesis is that sediment characteristics will differ in swamps from urbanised and intact catchments, and these differences will relate to differences in the microbial communities. Using co-extracted environmental total DNA and RNA we employed the molecular techniques of T-RFLP community fingerprinting, to characterise the microbial communities. While T-RFLP does not offer the detailed resolution of amplicon sequencing, it allows a comparable assessment of microbial community structure relative to environmental variables with greater replication with time and funding constraints [29]. Assessing the DNA and RNA provides insight into the presence, potential and activity of these genes and processes [30]. DNA degrades slowly in the environment and so represents active, inactive and deceased organisms. RNA degrades rapidly, and so reflects only the genes and species that have been recently active at the time of sampling [31]. We also measured gene abundances and transcription using quantitative PCR of the housekeeping 16S rRNA for bacteria and archaea to indicate the overall relative abundance and transcription/activity [32], and functional genes related to microbial metabolic carbon/methane cycling (*mcrA* and *pmoA*) and nitrogen cycling (Archaea *amoA*).

## Methods

### Study sites

**Fig 1:**
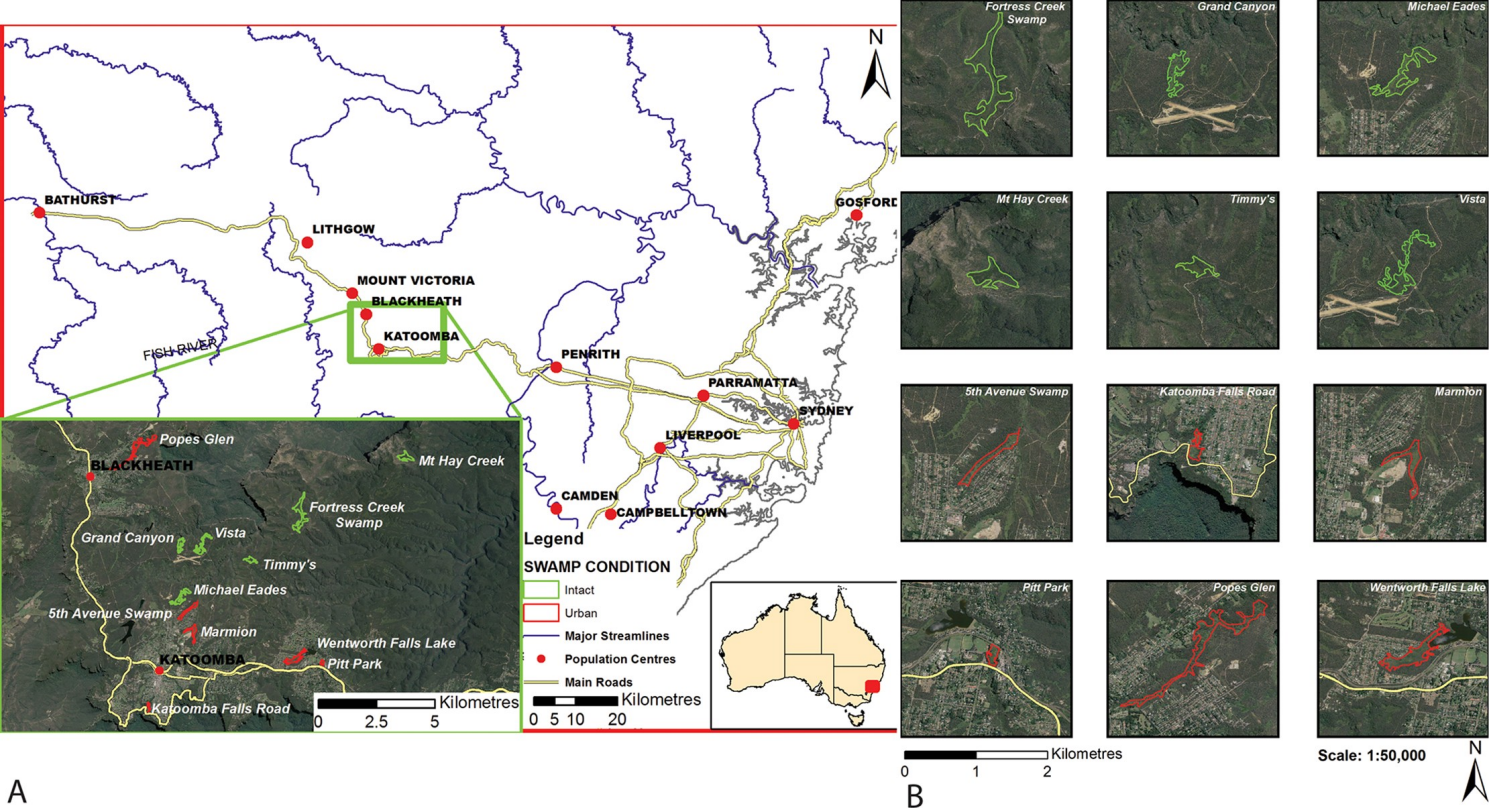
Study site locations. Red outlines are swamps in urbanised catchments and green outlines are swamps in intact catchments. Source: Basemaps produced with ArcGIS software by Esri. Sources: Esri, DigitalGlobe, GeoEye, Earthstar Geographics, CNES/Airbus DS, USDA, USGS, AeroGRID, IGN, and the GIS User Community. Swamp outlines taken from the Macquarie University, K Fryirs & G Hose 2016, THPSS mapping layer. 6 maps showing the spatial distribution of THPSS were produced for the following areas: Blue Mountains—VIS_ID 4480 Budderoo—VIS_ID 4481 Gosford—VIS_ID 4482 Newnes—VIS_ID 4483 Woronora—VIS_ID 4484 Penrose—VIS_ID 4485. Creative Commons license at: https://data.nsw.gov.au/data/dataset/temperate-highland-peat-swamps-on-sandstone-thpss-vegetation-maps-vis-ids-4480-to-4485.

The study area is approximately 100 km west of Sydney in the Blue Mountains (Fig 1). Within this area, low relief valley bottom swamps overlying sandstone (Temperate Highland Peat Swamps on Sandstone; THPSS) are common [33]. The township of Katoomba, located within the study area, receives a mean annual rainfall of 1403 mm. Mean maximum temperatures range from 9.4°C in July to 23.3°C in January and mean minimum temperatures range from 2.6°C in July to 12.9°C in February [34].

Twelve swamps were used in this study. Six ‘reference’ swamps that had intact catchments with natural vegetation, little to no impervious area (<15%), and no stormdrains were compared to six swamps that had catchments with varying degrees of urbanisation (Fig 1, Table 1). The urbanised catchment swamps were located within the townships of Katoomba, Wentworth Falls or Blackheath. Based on remote sensing data [12], the catchments of urban swamps have between 31.9% and 62.7% impervious area and between zero and eleven stormwater drains discharging into the swamp during and after rainfall events (Table 1). The intact catchment swamps had between zero and 13.1% impervious area (Table 1). Swamps were dominated by low sedge and surrounded by eucalypt woodland [35]. All but one of the urban swamps were channelised (i.e. contained a continuous, incised channel) and some had non-native plant communities, while all of the intact catchment swamps were non-channelised valley fills with predominantly native plant communities [36]. The geomorphic and sedimentological structure of the swamps have been described previously [13, 15, 37–39]. In brief, the sediment profile has distinct layers with unique properties important to the structure and function of the swamps. The surface layer, classified as surficial organic fines (SOF), is comprised of living and decomposing organic matter and fine silt and sand. The SOF layer goes to a depth of approximately 10–40 cm. In some cases, below the SOF layer there is a contemporary sand (CS) layer. CS are usually associated with erosion and disturbance. Below the SOF (and CS if present) is an organic and mineralised sediment layer classified as alternating organic sands (AOS). The AOS layer is typically the thickest sedimentary unit, often extending over a metre in thickness, and the location of the greatest amount of stored carbon [15, 37]. Below the AOS layer are fine cohesive sands (FCS) and basal sands and gravel (BSG) that sit atop saprolite and the sandstone bedrock. The thickness of each layer can vary between swamps and within a swamp [15, 37].

**Table 1:** Summary of environmental conditions of sampled swamps.

Catchment type	Swamp	Latitude, Longitude	Sample depth	Soil Moisture (%)	Organic Content (%)	Ammonium (mg/kg)	pH	EC (μS/cm)	Imperious Catchment (%)	Stormdrains (#)	Elevation (m)	Channellised
Intact	Fortress Creek	S33.6551, E150.365	0	229.7 (151.7)	26.1 (13.4)	2.6 (0.6)	4.6 (0.2)	80.3 (5.9)	0	0	860	N
			50	42.6 (24.9)	6.2 (6)	0.9 (0.3)	4.7 (0.2)	35.8 (18.5)				
	Grand Canyon	S33.664, E150.319	0	259.4 (189.7)	21.5 (14.3)	4.3 (1.6)	5 (0.3)	99.5 (41.9)	4.9	0	962	N
			50	46.9 (24.2)	5.8 (4.7)	0.9 (0.4)	4.8 (0.5)	24 (21.2)				
	Michael Eade's Reserve	S33.6843, E150.319	0	302.6 (12.2)	46 (3.6)	12 (1)	4.5 (0.2)	86.6 (47.5)	13.1	0	944	N
			50	27.7 (18.1)	3.5 (1.8)	1.8 (0.3)	4.6 (0.2)	35.3 (20.7)				
	Mt Hay Creek	S33.6289, E150.405	0	521.5 (338.1)	42 (17.5)	3.2 (0.7)	4.7 (0.4)	68 (13)	0	0	782	N
			50	57.2 (42.3)	7.6 (5.4)	2 (1)	4.4 (0.1)	67.1 (22.4)				
	Timmy's	S33.6686, E150.347	0	311 (138.3)	34.8 (14.2)	1.9 (3.1)	4.6 (0.1)	87.9 (10.5)	0	0	924	N
			50	81.3 (48.4)	21.9 (16.1)	1.6 (0.5)	4.5 (0.2)	42.5 (15)				
	Vista	S33.665, E150.328	0	221.1 (55.8)	31.9 (4.5)	5.9 (1.6)	4.6 (0)	87.8 (40.3)	0	0	957	N
			50	44.6 (15.7)	9.2 (4.7)	0.9 (0.7)	4.7 (0)	41.5 (27)				
Urbanised	Fifth Avenue	S33.6865, E150.324	0	170.3 (112.6)	19.5 (10.6)	7.6 (2)	5.4 (0.8)	49.8 (25.1)	44.3	4	920	Y
			50	61.6 (53.9)	7.4 (10.2)	2.8 (2.2)	5.4 (0.2)	25.3 (23.3)				
	Katoomba Falls Rd	S33.7258, E150.307	0	307.3 (43.4)	35.1 (13.9)	15 (8.1)	5.6 (0.2)	102 (14.5)	51.9	2	948	Y
			50	62.9 (34.8)	10.8 (6.7)	3.6 (2.3)	5.4 (0.3)	21.2 (12.9)				
	Marmion Rd	S33.6956, E150.325	0	135.9 (101.1)	19.8 (13.5)	6.8 (3.4)	4.9 (0.5)	110.3 (47.7)	51.6	0	943	Y
			50	28 (4.6)	4.8 (0.8)	3 (3.5)	5.1 (0.5)	26.1 (13.8)				
	Pitt Park	S33.7087, E150.374	0	201.8 (20.1)	19.1 (0.5)	10.7 (3.1)	5.9 (0.2)	94.4 (18.7)	31.9	8	872	Y
			50	120.5 (91.7)	10.4 (7.9)	2.8 (0.3)	6.2 (0.2)	45.5 (28.2)				
	Popes Glen	S33.6336, E150.293	0	177.1 (117.4)	23.7 (16.3)	5.3 (3)	6 (0.1)	85.2 (72.3)	38.5	6	1024	Y
			50	31.4 (10)	3.6 (2.1)	2.7 (1.6)	5.8 (0.1)	30.3 (24.7)				
	Wentworth Falls Lake	S33.7077, E150.362	0	462.6 (19.4)	47 (8.8)	8.3 (2.7)	5.9 (0.2)	75.5 (41.4)	62.7	11	893	N
			50	61.1 (2.2)	6.3 (2.3)	4.6 (3)	5.3 (0.2)	23.8 (12.8)				

Sediment condition measures presented are averages (n = 3), and numbers in parenthesis are standard deviation. Impervious catchment and stormdrain data are from [12].

### Sample collection

Sampling occurred at three locations along the central axis of each swamp using a Russian D-corer. Sediment samples were collected at the surface (top 1–2 cm) to target the SOF layer and at a depth of 50 cm to target the AOS layer or CS layer if present. The SOF layer is likely to have greater oxygen availability favouring aerobic microbial metabolic processes, and the AOS or CS at 50 cm depth is more mineralised and, being deeper in the sediment profile, is more likely to favour anaerobic microbial metabolic processes [6]. At the time of sampling, the sediment type and the vegetation community was noted and photographed. Of our 36 deep samples, 9 were CS and all but one of these were from urbanised catchment swamps. Sediment for molecular and chemical analysis was collected into separate tubes (2 ml Eppendorf for molecular and 50 ml Falcon centrifuge tubes for sediment analysis) and snap-frozen immediately using dry ice and stored at -80° C and -20° C, respectively.

### Sediment analysis

Sediments were analysed for electrical conductivity, pH, ammonium and nitrate concentrations as potential indicators of pollution and stormwater runoff [11, 16] and soil moisture and organic content that we hypothesised would be potentially effected by altered water tables and erosion associated with urbanisation [12]. Electrical conductivity and pH was measured in a 1:1 mass ratio sediment/deionized water slurry using an electrical conductivity meter (Eutech Instruments Pte Ltd/Oakton Instruments Eutech Cyber scan CON 400) and pH meter (Thermo Scientific Orion 3 star pH meter). Ammonium and nitrate content was measured in sediment extract using APHA Standard Methods for the Examination of Water and Wastewater by the National Association of Testing Authority (NATA), accredited Sydney Analytical Laboratories, Seven Hills, NSW. Soil moisture was measured gravimetrically after drying overnight (105° C for >12 h) and total organic content was determined by loss on ignition (550° C for 5 hrs) [40]. Soil moisture and organic content were calculated as a percentage of dry weight.

### Molecular analysis

#### Extraction

RNA and DNA were co-extracted from sediment samples within one week of collection using the PowerSoil Total RNA Isolation Kit and DNA Elution Accessory Kit (MoBio) according to the manufacture’s protocol. Genomic DNA was eliminated from purified total RNA using the Isolate II RNA mini Kit (Bioline) with on-column DNase digestion. Purity and yield of purified nucleic acids was quantified using a NanoDrop 2000 spectrophotometer (Thermo Scientific Inc.). For each sample, 100 μg of total RNA was converted into cDNA with Tetro cDNA Synthesis Kit (Bioline). DNA and cDNA was diluted with MilliQ water to avoid PCR inhibitors (1:30 cDNA and 1:150 DNA). Hereafter the RNA product of cDNA will be referred to as RNA or transcriptions in the text.

#### Terminal restriction length polymorphism (T-RFLP)

Terminal restriction length polymorphism (T-RFLP) analysis was used to compare community structure of bacteria and archaea between sites, catchment types and depth within the sediment profile. The bacteria and archaea 16S rRNA gene was PCR amplified from DNA and RNA samples using FAM-labelled universal primers (Table 2) and MyTaq polymerase (Bioline). PCR amplicons were digested with restriction enzymes (Table 2). PCR conditions were as follows: 94°C for 2 min then 35 cycles of 94°C for 15 sec, annealing temperature for 30 sec and 72°C for 2 min. Digested PCR products were analysed on ABI3730xl Genetic Analyser at the Australian Genome Research Facility with LIZ1200 size standard. Data were processed using GeneMapper Software and the online tool T-REX [41]. Peak noise was removed [42], peaks were aligned within two base pairs and peaks that occurred only once in the dataset were removed.

**Table 2:** PCR primers, conditions and references for terminal restriction length polymorphism (T-RFLP).

Gene	Name	Function	Sequence 5' - 3'	Annealing (°C)	Restriction enzyme(s)	Reference
16S rRNA Bacteria	27F	F primer*	AGAGTTTGATCCTGGCTCAG	54	HhaI	[43]
	1492R	R primer	TACCTTGTTACGACTT			
16S rRNA Archaea	1Af	F primer*	TCYGKTTGATCCYGSCRGAG	53	HhaI, Sau96I	[44]
	1100AR	R primer	TGGGTCTCGCTCGTTG			

* FAM labelled

#### Gene abundance and transcription abundance (qPCR)

Gene abundances (DNA) and transcription abundance (RNA) were estimated using quantitative PCR (qPCR). A TaqMan qPCR assay was used to quantify the archaea and bacteria 16S rRNA gene using universal primer pairs (Table 3) and a FAM-labelled probe with SensiFast Probe mix (Bioline) in 25 μL reactions. SYBR green qPCR assay was used to quantify the functional genes for methane production (mcrA), methane oxidation (pmoA) and AOA archaea ammonia monooxygenase (amoA) using universal primers (Table 3) in 8 μL reactions using SensiFast SYBR No-ROX (Bioline). All qPCR reactions were carried out on a BioRad CFX96 RT System C1000TM Thermal Cycler. PCR conditions were as follows: initial denaturation (94°C, 2 min) followed by 40 (TaqMan/SYBR analysis) cycles of denaturation (94°C, 5 sec) and hybridisation-elongation (annealing temperature, 45 sec). A subsequent melting temperature curve of the amplicon was performed in the SYBR green qPCR assays.

**Table 3:** The primers, conditions used for the quantitative PCR.

Gene	Name	Function	Sequence 5' - 3'	Annealing (°C)	Reference
*pmoA*	A189gc	F primer	GGNGACTGGGACTTCTGG	62	[45]
	mb661	R primer	CCGGMGCAACGTCYTTAC		[46]
AOA *amoA*	AamoAF	F primer	STAATGGTCTGGCTTAGACG	60	[47]
	AamoAR	R primer	GCGGCCATCCATCTGTATGT		
*mcrA*	ML-F	F primer	GGTGGTGTMGGDTTCACMCARTA	60	[48]
	ME-2	R primer	TCATKGCRTAGTTDGGRTAGT		[49]
16S rRNA Archaea	ARC787F	F primer	ATTAG ATACC CSBGT AGTCC	60	[32]
	ARC915F	TaqMan*	AGGAA TTGGC GGGGG AGCAC		
	ARC1059R	R primer	GCCAT GCACC WCCTC T		
16S rRNA Bacteria	BAC338F	F primer	ACTCC TACGG GAGGC AG	60	[32]
	BAC516F	TaqMan*	TGCCA GCAGC CGCGG TAATA C		
	BAC805R	R primer	GACTA CCAGG GTATC TAATC C		

* FAM and TAMRA labelled.

The presence of targeted genes was confirmed with electrophoresis gel and analysis of the melting curve data. Calculations of amplicon copy numbers were based on serial dilutions of target genes cloned into the pCR4-TOPO vector (Thermo Scientific Inc.), standard curves all with r^2^ ≥ 0.99 and dynamic range of 1e^2^ – 1e^8^ copies. For each qPCR run, negative controls were included to confirm the integrity of the results [50].

### Statistical analysis

Swamps were considered the replicates, so multiple values collected per swamp were averaged to provide a single value. Sediment and catchment properties and gene abundances were compared using a 3-factor repeated measures analysis of variance (ANOVA). In these analyses, catchment type (between-subject factor) and depth (within-subject factor) were considered fixed factors and site was considered a random factor and nested within catchment type. Data were assessed for normality using Q-Q plots and log transformed where necessary to meet this assumption. Sphericity was tested using Mauchley’s Test and the Geisser-Greenhouse Adjustments used where the assumption was not met. Tukey’s post hoc pair-wise comparisons were used to test for differences between levels where there was a significant interaction.

Gene abundance data were log transformed to approximate normality. Relationships between the sediment variables, gene abundances and measures of urbanisation (impervious catchment and stormwater drains entering swamp) were tested using Pearson correlation analysis. The above analyses were conducted in NCSS version 10.0.7.

Assemblage data based on T-RFLP profiles were visualised using non-metric multidimensional scaling (nMDS). Relationships between assemblages and environmental variables were visualised using distance based redundancy analysis (dbRDA [51]) and distance based linear models (DistLM) using stepwise selection. Distance-based redundancy analysis is a constrained ordination method similar to Redundancy Analysis, which allows the use of non-euclidean distance measures, here, Bray-Curtis. Peak height data were standardised (by total sample peak area) and square root transformed [52] prior to analysis. The abiotic data were normalised and checked for strong correlations with draftsman plots. Variables with a correlation coefficient greater than 0.9 were removed from subsequent analyses [53].

The comparison of assemblages between catchment types and depths was done using PERMANOVA, with a 3-factor linear model replicating that of the repeated measures ANOVA described above. Multivariate analyses were done using PRIMER & PERMANOVA + add on version 1.0.8 (PRIMER-E Ltd.) using the Bray-Curtis similarity index. The significance level (α) for all analyses was 0.05 and was adjusted, where necessary, to account for multiple comparisons using the Holm-Bonferroni method [54].

## Results

### Sediment characteristics

**Fig 2:**
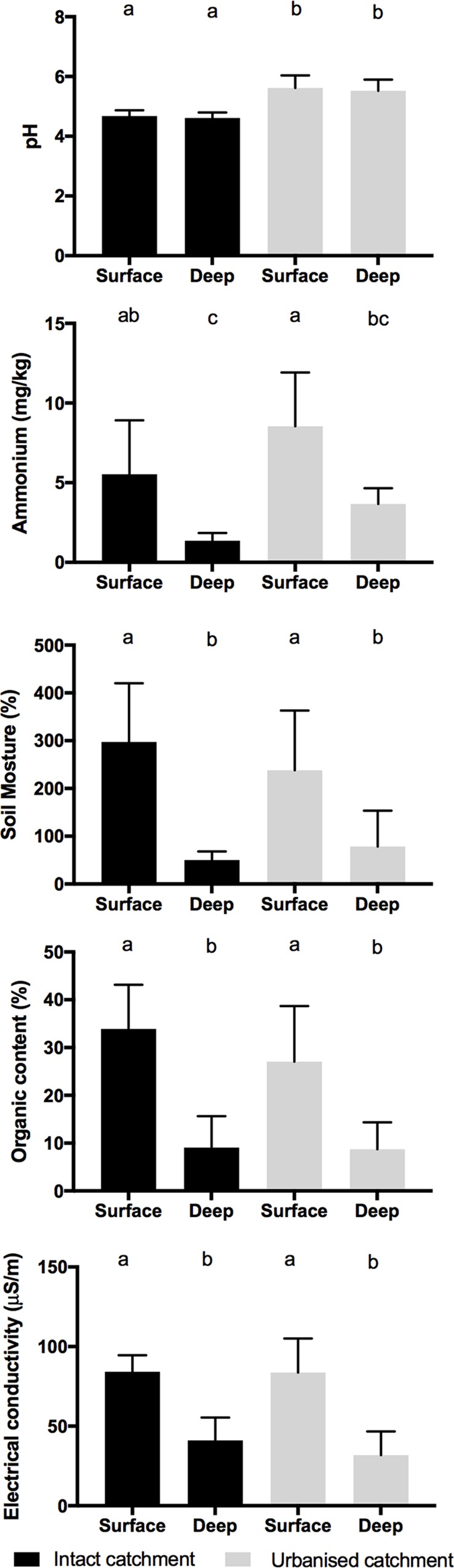
Sediment parameters by catchment type and depth. Mean (± Std dev) sediment quality parameters from surface (0 mm) and 50 cm depth in swamps with urbanised and intact catchments (n = 6). Different letters indicate statistical differences (p<0.05).

Soil pH varied by catchment type (p<0.001) with significantly higher pH (mean 5.6) in urbanised catchments. There was no difference in soil pH with depth nor was the interaction significant (p>0.05). Soil ammonium concentrations were significantly (p = 0.003) higher in the swamps of urbanised catchments (Table 1, Table 4, Fig 2) compared to swamps with intact catchments, and also higher in surface sediments than in deep sediments (p<0.001), whereas the depth x catchment interaction was not significant.

**Table 4:** Summary of sediment conditions by catchment type.

Catchment type	Sample depth	Soil Moisture (%)	Organic Content (%)	Ammonium (mg/kg)	pH	EC (μS/cm)
Intact	0	308 (184)	33.7 (13.6)	4.98 (3.75)	4.67 (0.26)	85.0 (28.2)
	50	50.1 (31.3)	9.05 (9.07)	1.35 (0.67)	4.61 (0.29)	41.0 (22.3)
urbanised	0	243 (134)	27.4 (14.6)	8.93 (4.82)	5.60 (0.53)	86.2 (40.5)
	50	60.9 (49.6)	7.20 (5.79)	3.25 (2.11)	5.53 (0.44)	28.7 (18.9)

Sediment condition measures presented are mean (n = 18), and numbers in parenthesis are standard deviation.

The remaining abiotic variables, organic content, soil moisture content, and electrical conductivity did not vary by catchment type (p>0.31), but all varied by depth. There were no significant depth x catchment type interactions (p>0.29). The organic content, moisture content and electrical conductivity were all significantly (p<0.001) higher in the surface than deep sediment (Table 1, Table 4, Fig 2). Nitrate concentrations were below the method detection limit of 0.1 mg/kg, with three exceptions, two 0.1 mg/kg (Wentworth Falls Lake a surface and a deep sample) and one 0.2 mg/kg (Grand Canyon surface sample). Because nitrate was detected in so few samples and only in very low concentrations it was not included in subsequent analyses.

Soil pH was significantly correlated with catchment impervious area and the number of stormwater drains entering the swamp, in both shallow and deep soils (Table 5). Ammonium concentrations in deep soils were correlated with catchment impervious area and the number of stormwater drains, but not in shallow soils (Table 5). Other sediment properties were not significantly correlated with either the measure of urbanisation (Table 5).

**Table 5:** Pearson correlation coefficient for significant relationships between sediment characteristics and measures of urbanisation and gene and transcription abundances. Values presented are statistically significant (p<0.05).

				DNA				RNA	
Depth (cm)		Impervious Catchment Area (%)	Number of Stormwater Drains	Bac 16S	Arc 16S	*pmoA*	*mcrA*	Bac 16S	Arc 16S
0	pH	0.75	0.87	0.81	0.77	0.77	ns*	0.82	0.83
	EC (μS/cm)	ns	ns	ns	ns	ns	ns	ns	ns
	Ammonium (mg/kg)	ns	ns	ns	ns	ns	ns	ns	ns
	Soil Moisture (%)	ns	ns	ns	ns	ns	ns	ns	ns
	Organic Content (%)	ns	ns	ns	ns	ns	ns*	ns	ns
50	pH	0.77	0.74	ns	ns	ns	ns	ns	ns
	EC (μS/cm)	ns	ns	ns	ns	ns	ns	ns	ns
	Ammonium (mg/kg)	0.78	0.75	ns	ns*	ns	ns	ns	ns
	Soil Moisture (%)	ns	ns	ns	ns	ns	ns	0.74	ns
	Organic Content (%)	ns	ns	ns	ns	ns	ns	ns	ns

Pearson’s coefficients for correlations between sediment properties in swamps and indicators of catchment urbanisation and gene abundance. EC = electrical conductivity. ns = non-significant (p>0.007) following Holm-Bonferroni Correction

*Pearson correlation coefficient significant prior to Holm-Bonferroni Correction (α = 0.05).

Among the sediment properties, sediment moisture and organic content were strongly correlated (r = 0.91). Consequently, soil organic content was excluded from subsequent multivariate analyses (see below). Correlations among other soil variables were <0.8.

### Microbial community analysis (T-RFLP)

#### Bacterial community (DNA)

**Fig 3:**
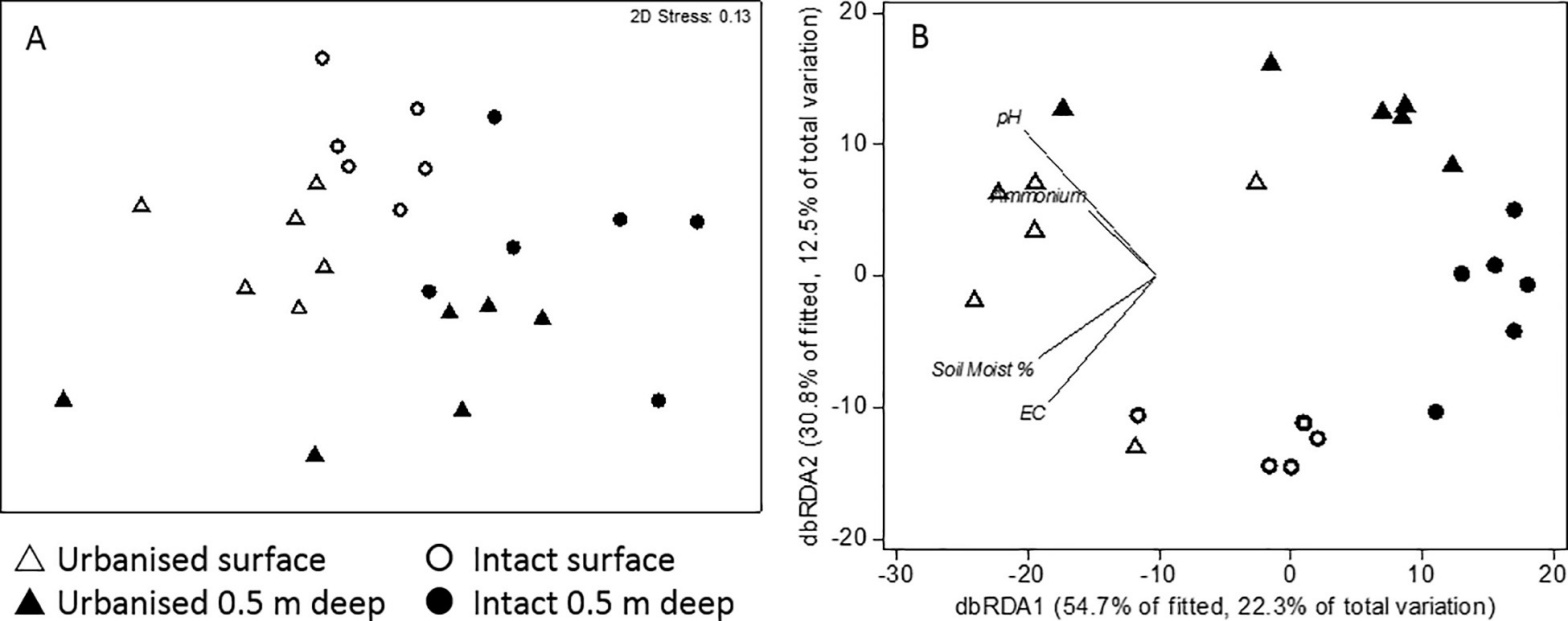
nMDS plot of bacteria DNA community. Communities from intact catchment swamps are indicated by circles and those from urbanised catchments marked triangles. Open symbols represent surface samples and closed symbols represent samples from 50 cm depth. Soil Moist % = soil moisture as % dry weight, EC = electrical conductivity (μS/cm) and ammonium = ammonium concentration (mg/kg).

The bacterial community composition (DNA) differed significantly by catchment type (PERMANOVA, p = 0.001, r^2^ = 0.12) and depth (p = 0.001, r^2^ = 0.18) and but their interaction was not significant (p = 0.218, r^2^ = 0.04). This difference is evident as clear separation of surface and deep samples of urbanised and intact swamps in the nMDS ordination (Fig 3A).

When tested alone, all variables were significantly correlated with microbial community structure (r^2^ = 0.12–0.17, S1 Table), but stepwise selection identified only electrical conductivity and pH as explaining a significant and unique proportion of the variation in the bacterial community composition (DistLM, r^2^ = 0.35). The addition of other variables did not increase significantly the variation explained by the stepwise model. As suggested by the analysis of the abiotic variables above, pH was strongly correlated with catchment type whereas electrical conductivity was correlated with sample depth.

#### Transcribing bacterial community (RNA)

**Fig 4:**
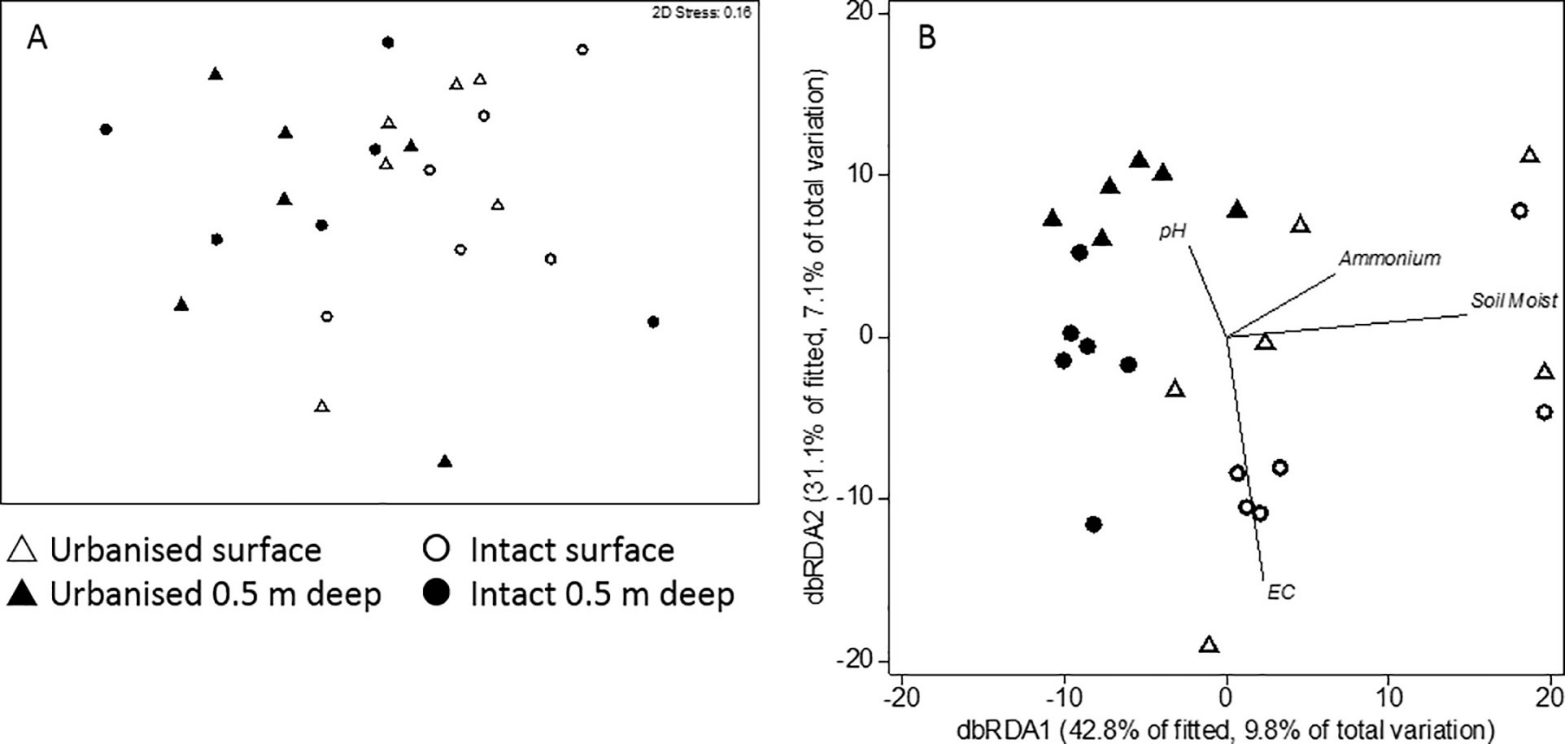
nMDS and dbRDA ordination plots of bacteria RNA community. Communities from intact catchment swamps are indicated by circles and those from urbanised catchments marked triangles. Open symbols represent surface samples and closed symbols represent samples from 50 cm depth. Soil Moist % = soil moisture as % dry weight, EC = electrical conductivity (μS/cm) and ammonium = ammonium concentration (mg/kg).

The metabolically active bacterial community (RNA) differed significantly between depths (p = 0.009, r^2^ = 0.10), but this separation was not clear in the nMDS ordination (Fig 4A). There was no significant difference in assemblages between catchment types (p = 0.526, r^2^ = 0.04), nor was the catchment type x depth interaction significant (p = 0.781, r^2^ = 0.02). The difference in assemblages with depth is evident in Fig 4B, with deep samples to the left of the figure and shallow samples to the right. This separation is correlated is with differences in soil moisture, as indicated by the vector in that same direction.

When tested alone, soil moisture and electrical conductivity were significantly correlated with microbial community structure (r^2^ = 0.09, 0.08, respectively, p≤0.025, S1 Table), while ammonium and pH were not (p>0.06). Stepwise selection identified only soil moisture as explaining a significant and unique proportion of the variation in the bacterial community composition (DistLM, r^2^ = 0.09) and the addition of electrical conductivity, pH and ammonium did not increase significantly the variation explained by the model (S1 Table).

#### Archaeal community (DNA)

A number of the samples did not PCR amplify the archaea 16S rRNA gene sufficiently for T-RFLP analysis (S2 Table). As a consequence, we only had T-RFLP profiles for surface soils from 2 of the 6 intact sites and no depth sample from VS (intact swamp). Despite the limited sampling of intact sites, the archaeal communities from intact and urbanised catchment sites were significantly different (p = 0.045, r^2^ = 0.10) although depth (p = 0.619, r^2^ = 0.03) and the interaction term (p = 0.343, r^2^ = 0.06) in the PERMANOVA were not significant.

**Fig 5:**
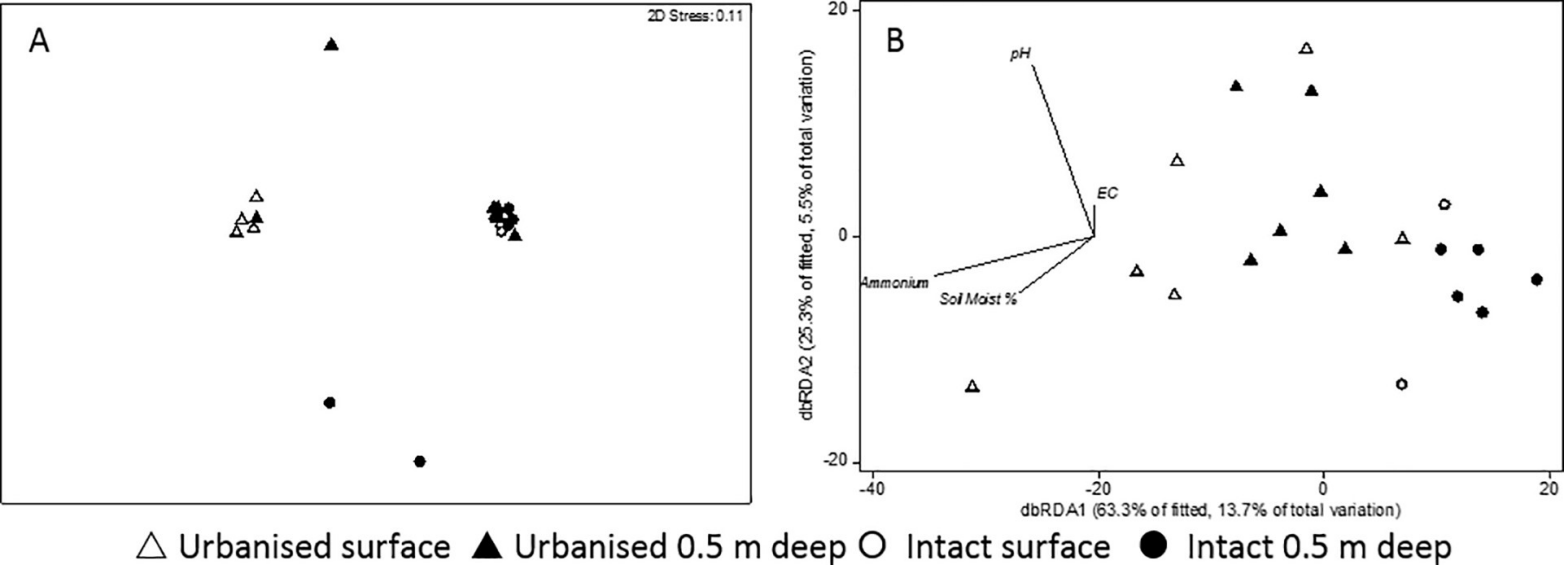
nMDS and dbRDA ordination plots of archaeal DNA community. Communities from intact catchment swamps are indicated by circles and those from urbanised catchments marked triangles. Open symbols represent surface samples and closed symbols represent samples from 50 cm depth. Soil Moist % = soil moisture as % dry weight, EC = electrical conductivity (μS/cm) and ammonium = ammonium concentration (mg/kg).

Differences in archaeal community structure were not clearly evident in the nMDS ordination (Fig 5A) but a separation is evident in Fig 5B, in which samples from intact and urbanised sites separate along the x axis, which is correlated most strongly with ammonium and soil moisture. Similar separations of samples by catchment type was evident in the nMDS and dbRDA ordinations when only deep samples were analysed (data not shown). The archaeal community structure was not significantly correlated with any of the environmental variables (DistLM, p>0.05).

#### Transcribing archaeal community (RNA)

Only seven surface and seven deep samples amplified and provided T-RFLP profiles (S2 Table). Only one of the surface samples and three of the deep samples were from an intact catchment swamps, which did not represent the variation across our sites, so these data were not analysed further.

### Gene abundances qPCR

#### Gene abundance (DNA)

**Fig 6:**
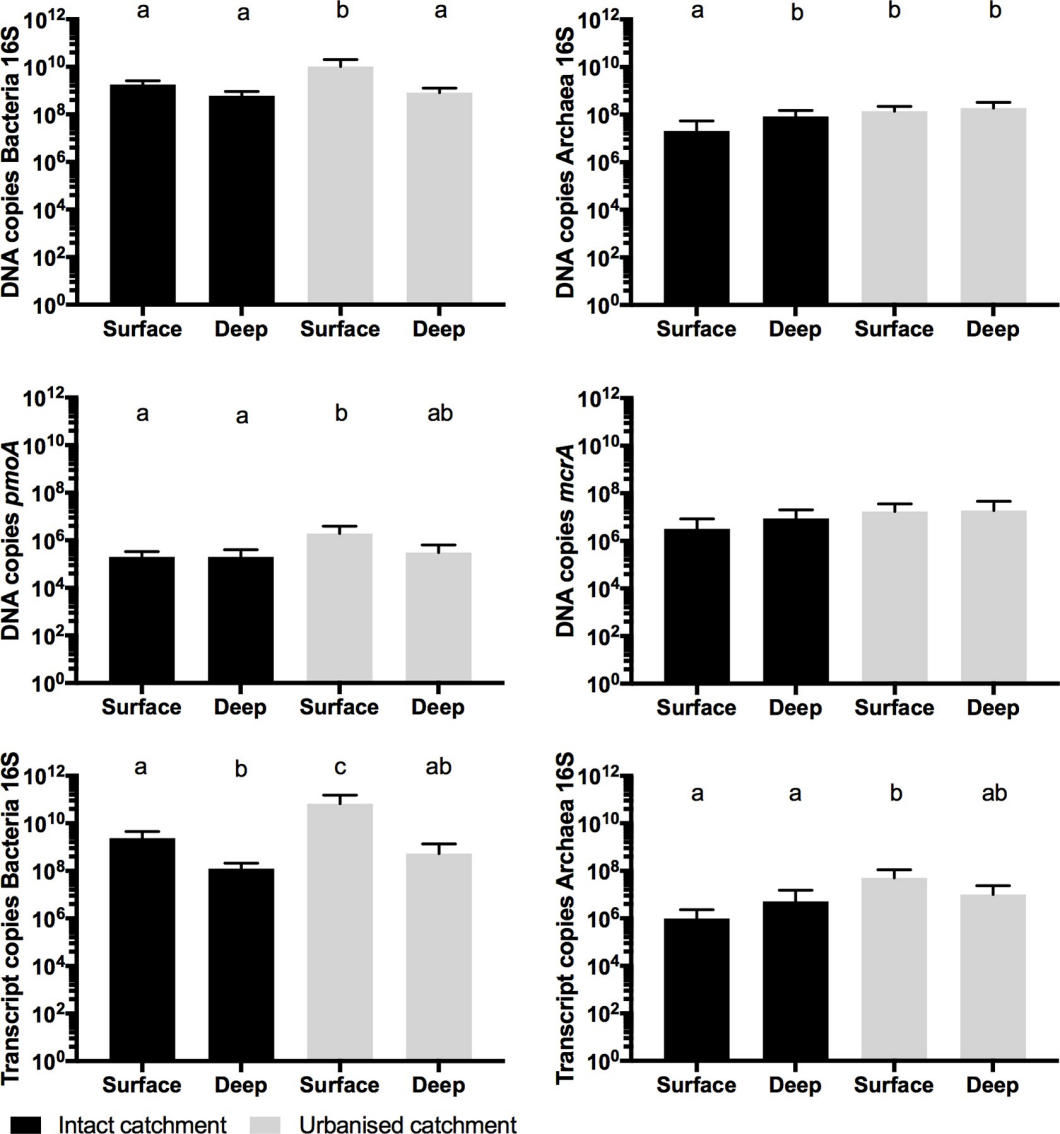
Gene and transcription abundances by catchment and depth. Mean (± Std dev) number of DNA/RNA gene copies in sediment collected from the surface and at depth (50 cm) in swamps with urbanised and intact catchments. (n = 6).

The bacterial 16S rRNA gene was significantly more abundant in the urbanised catchment swamps than the intact catchment swamps (p = 0.004) and more abundant in the surface samples than the deep samples (p<0.001), but the interaction was not significant (p = 0.100). The archaea 16S rRNA gene abundance varied by both catchment type (p = 0.003) and depth (p = 0.020) and the interactions between these factors was also significant (p = 0.044), indicating that the variation with depth was not consistent across catchment types. The pair-wise comparisons showed significantly lower abundance of the archaea 16S rRNA gene in the intact catchment surface samples than in the urbanised catchment samples and intact catchment deep samples (Table 6, Fig 6).

**Table 6:** Gene and transcription abundances summary by catchment and depth.

Catchment type	Sample depth	RNA Bacteria 16S rRNA	RNA Archaea 16S rRNA	DNA Bacteria 16S rRNA	DNA Archaea 16S rRNA	DNA *mcrA*	DNA *pmoA*
Intact	0	2.36E+9 (4.12E+9)	1.28E+6 (2.66E+6)	1.78E+9 (1.33E+9)	2.08E+7 (5.78E+7)	3.38E+6 (8.76E+6)	2.02E+5 (1.70E+5)
	50	1.24E+7 (1.88E+8)	6.86E+6 (2.04E+7)	6.12E+8 (6.63E+8)	8.33E+7 (8.91E+7)	1.39E+7 (1.55E+7)	2.81E+5 (2.27E+5)
urbanised	0	6.64E+10 (1.20E+11)	5.04E+7 (8.76E+7)	1.04E+10 (1.12E+10)	1.39E+8 (1.47E+8)	1.93E+7 (2.81E+7)	2.04E+6 (2.60E+6)
	50	4.56E+08 (7.68E+8)	9.86E+6 (1.80E+7)	7.78E+8 (1.07E+9)	1.72E+8 (2.03E+8)	1.85E+7 (2.90E+7)	3.25E+5 (6.10E+5)

Summary of qPCR results summary as mean number of copies per gram of sediment. Numbers in parenthesis are standard deviation.

The abundance of the *pmoA* genes was significantly lower (p = 0.037) in samples from intact swamps than in those from urbanised swamps, while there was no difference in gene abundance with depth (p = 0.126), nor was the interaction significant (p = 0.086, Fig 6). The abundance of *mcrA* genes did not vary between catchment types (p = 0.088) or depth (p = 0.957), nor was the interaction significant (p = 0.802). The ammonia oxidizing archaea *amoA* gene had the lowest detection rate only being detected in 15 out of 71 samples. Since so few samples had detections we did not compare abundances, however it is worth noting that 12 (80%) of the archaea *amoA* detections were from urbanised swamps.

#### Gene transcription abundance (RNA)

The bacteria 16S rRNA gene transcription was significantly more abundant in swamps with urbanised catchments than in those with intact catchments (p = 0.016), and in surface compared to deep samples (p = <0.001). The interaction of these factors was not significant (p = 0.101, Fig 4). Archaea 16S rRNA gene transcription abundances were also greater in urban than in intact catchment swamps (p = 0.016, Fig 4) but did not differ with depth (p = 0.366). The interaction of depth and catchment type was also not significant (p = 0.139).

We were not able to detect the functional gene transcription numbers in most of the samples, which suggests low or no activity of these genes. Although we were not able to compare transcription abundances, there were patterns in the relative frequency of detections. For example, the methanogen *mcrA* gene transcription was detected at 50% of the intact catchment swamps and in all but one of the urbanised catchment swamps, however, the reasons for this heterogeneity are unclear. In both catchment types, *mcrA* was more frequently detected in the deeper samples (unpooled samples: intact 6/18, urbanised 10/16) than the surface samples (unpooled samples: intact 3/18, urbanised 7/18). The *pmoA* gene transcription was also more frequently detected in the samples from urban catchment swamps (6/6 surface and 4/6 deep) than in the samples from intact catchment swamps (4/6 surface and 0/6 deep).

### Sediment characteristics influence on community structure

None of the abiotic variables had correlations with each other exceeding 0.9, Therefore all of the environmental variables were included in the subsequent analyses. The strongest correlation was between organic content (%) and soil moisture content (%) (r = 0.88). The correlations between the remaining variables were all less than 0.5.

#### Bacterial community (DNA)

The influences of the abiotic variables on the microbial community (DNA) modelled by DistLM indicated that pH had the greatest influence at both depths. In the surface sediments, pH was the only variable to explain a significant proportion (22%) of the variation in community structure (Fig 4). In the deep sediments, pH (12.9%), electrical conductivity (8.9%) and organic content (1.6%) were significant (p<0.05) in explaining the variation in community structure.

#### Bacterial transcription community (RNA)

In the surface sediments, pH was the only variable to explain a significant proportion of the variation in the transcribing community (8.7%). In the deeper samples none of the environmental variables were significant in explaining the variation.

#### Archaeal community (DNA)

Environmental variables did not explain a significant proportion of the variation in community structure in either the surface or the deep sediments. In part, this may be due to the low number of samples that had enough PCR product for analysis (11 surface samples and 21 deep samples each out of 36 total samples). The number of RNA samples were 7 for each the surface and deep communities, out of a total of 36 of each.

### Sediment characteristics influence on gene abundance and transcription

There was a significant positive correlation (p<0.05) between the pH and the abundance of each of the genes except *mcrA* measured in the surface sediment (Table 5) however, no other genes or variables in the surface sediments were significantly correlated (p>0.05). In the deep sediments, the abundance of the archaea 16S RNA were negatively correlated with both sediment organic content and moisture. No other genes or variables in the deep sediments were significantly correlated.

AOA *amoA* (ammonia oxidising archaea) was only detected in 21% of samples and therefore was not included in the correlation analysis. It is worth noting, however, that sediments in which AOA *amoA* was detected had above average pH values (5.51 versus 5.1) and ammonium (7 versus 4.6 mg/kg) concentrations.

## Discussion

The upland swamps in the World Heritage-listed Blue Mountains appear to be affected by catchment urbanisation. Our results suggest that urbanisation is impacting the microbial community and may be impacting the subsequent capacity for delivering ecosystem services, as well as changing water and sediment quality. Increases in the pH of stream water are a common consequence of catchment urbanisation [17], and, not surprisingly, pH was strongly and positively correlated to the number of stormwater drains entering the swamp and the impervious catchment area in this study (Table 5). The sediment pH was on average about 1 pH unit (ten times H^+^ ion concentration) higher in urbanised catchment swamps than in intact catchment swamps (4.6 ±0.27 vs. 5.7±0.48), which agrees with previous studies of swamp surface [11, 14, 55] and pore waters [6]. An increase in pH is not surprising considering naturally acidic waters, such as in these catchments, cause dissolution of calcium, bicarbonate and potassium ions from concrete from urban infrastructure and storm drains, thereby increasing the alkalinity and pH of waters [17, 18]. Nutrient levels typically increase within urbanised catchments [16], and we also found that ammonium levels were correlated to the measures of urbanisation. Although we had measureable levels of ammonium, nitrate was generally below detection limits. This suggests that the nitrate in the swamps, which are naturally nutrient poor [36, 56], is being lost through leaching, microbial cycling and denitrification, or by plant uptake [57].

The elevated pH that is associated with an urbanised catchment, may affect how microbial assemblages cycle and store carbon. Less acidic/more neutral pH [58] and increased nutrients [59, 60], have both been linked to higher carbon mineralisation in peatlands. Globally, peatlands store approximately a third of global carbon and are also responsible for the greatest natural source of methane emissions [60–62]. Small perturbations of the natural state of peatlands can shift these ecosystems from carbon sinks to net greenhouse gas sources [25, 63]. In general, these east coast Australian peat forming swamps differ from high latitude northern hemisphere peatlands, in that they experience significant inter-annual climatic variation [64] resulting in variable hydrology [38] and marginal, localised and varied peat formation [37]. Carbon cycling, both accretion and storage, in peatland swamps is controlled by complex interactions between the plants, soil conditions and the microbes present [65, 66]. The upland swamps of eastern Australia are most similar to valley mire fens and tend to be naturally nutrient poor, groundwater and rainwater fed, dominated by sedges and mixed species [36, 38, 39] and have a natural pH between 4 and 5 (Fig 2) [6, 14, 36, 56]. Sedge dominant fens tend to mineralise carbon, produce more methane and cycle nutrients more rapidly than sphagnum dominated peat swamps [66]. The study swamps are marginally peat-forming [37] and so, altered conditions may have profound impacts on the microbial communities and carbon cycling and storage in these swamps.

Acidic conditions in sediments restrict the species of bacteria present to those adapted to metabolizing in acidic conditions [67]. Indeed, we found that the transcription and abundance of the16S rRNA genes positively correlated with pH (Table 5). This is also shown by catchment type. The more pH-neutral urbanised catchment swamps had increased transcription of the bacteria and archaea16S rRNA gene compared with the more acidic intact catchment swamps (Fig 4). Using the 16S rRNA gene as a general estimate for relative bacteria and archaea abundance and activity [68], suggests surface sediments in urbanised catchment swamps had more archaea and the archaea and bacteria were more active generally than in intact catchment swamps. The potential implication of this greater microbial activity may be increased organic material breakdown and carbon export [69], which could result in decreased peat storage and formation in the urbanised catchment swamps.

The higher pH in swamps with urbanised catchments could also increase methane production. Although acid adapted methanogens exist, it is at circum-neutral pH that methane production is optimised [22, 70] and the greatest diversity of methanogens can be found [71]. We did not find significant differences in the abundance of the methanogen gene (*mcrA)* or methanotroph gene (*pmoA*) by catchment type, however *pmoA* did have strong positive relationships with increases in pH (Table 5), and *mcrA* was significant at p<0.05, but not when the correction was applied. Others have found a relationship between pH and methanogens elsewhere [71]. We were unable to statistically test the abundances of the transcription copies for *mcrA* and *pmoA* against sediment conditions or by catchment type, however the positive detection rates by catchment type suggest that *mcrA* and *pmoA* are more likely to be transcribed in the urbanised catchment swamps than in swamps with intact catchments. Transcription for the methanogen *mcrA* gene was detected about twice as frequently in urbanised catchment swamps compared with intact catchment swamps (50% vs. 28% detection rate). The *pmoA* gene was only detected in a single sample from an intact catchment swamp while it was detected in 11 urbanised catchment swamps samples. Transcription of the *pmoA* is likely to mean a reduction in the net methane production, but it does also indicate that methane is available for metabolic processes and is being produced [65].

Swamps in the Blue Mountains that have become channelized tend to be those most affected by urbanisation [12]. These swamps export up to 18 times more carbon than swamps that are not channelised [6]. Further, carbon dioxide and methane emissions account for 0.1% and 0.001% of the total carbon export in non-channelised swamps, respectively, whereas carbon dioxide and methane make up 19% and 0.06% of the carbon exported from channelised swamps [6]. Although much of the increase in carbon export is attributed to the geomorphological changes that occur with swamp channelization (5,60), it is ultimately a combination of physical, microbial and chemical factors, and feedbacks between them that results in greater carbon export [6].

The bacterial community analysis further supports the hypothesis that changes in pH associated with urbanisation are affecting the microbial function of these swamps, particularly in the surface sediment. Although we detected community differences by catchment type and that increased pH is a likely consequence of urbanisation (as discussed above), the pH was a stronger influence on community structure than was the catchment-scale changes in imperviousness. Other wetland studies have found that pH is a strong driver of bacterial community composition and diversity over large scales [20, 72]. Indeed, pH was a stronger determinant for microbial community than the geographic location, wetland type, nutrient or soil carbon levels, which is also consistent with previous studies [19, 20].

Understanding how urbanisation affects the microbial community is imperative to reducing impacts to swamps affected by urbanisation. Our results suggest that a more natural, acidic sediment pH (pH < 5) is important for maintaining the microbial community and swamp function. As pH increased, so did the abundance of archaea and bacteria16S rRNA gene, *mcrA* and *pmoA*, which may contribute to the augmented carbon cycling and methane production that has been observed in these swamps [6]. Given the link between elevated pH and stormwater infrastructure and impervious area, it is essential to continue to reduce these impacts. In addition to current strategies to minimize flash stormwater flows, using materials not prone to dissolution by naturally acidic waters may help maintain more natural pH levels [18].

## Supporting information

S1 Table Relationship between environmental variables and microbial assemblages as determined by T-RFLP of 16S genes in upland swamps of the Blue Mountains, NSW, Australia. Marginal tests indicate the relationship between environmental variables and assemblages individually. The sequential test indicates the relationship between environmental variables and assemblages determined by stepwise multiple regression. Partial R2 values indicate the relationship of a variable once those listed above it have already been fitted to the stepwise model. Bold indicates p < 0.05.
(DOCX)

S2 Table Summary of T-RFLP amplification. Number of samples out of a total 3 replicates that had sufficient amplification for analysis of DNA and RNA transcribing community analysis (T-RFLP).
(DOCX)
