# Supplementary material for: The impact of urbanisation on community structure, gene abundance and transcription rates of microbes in upland swamps of Eastern Australia
Source: PLoS One. 2019 Mar 4;14(3):e0213275. doi: 10.1371/journal.pone.0213275 (PMC6398846; doi:10.1371/journal.pone.0213275)
Supplement: S1 Table — Marginal tests indicate the relationship between environmental variables and assemblages individually. The sequential test indicates the relationship between environmental variables and assemblages determined by stepwise multiple regression. Partial R2 values indicate the relationship of a variable once those listed above it have already been fitted to the stepwise model. Bold indicates p < 0.05. (DOCX) [file pone.0213275.s001.docx]

S1 **Table. Relationship between environmental variables and microbial assemblages as determined by T-RFLP of 16S genes in upland swamps of the Blue Mountains, NSW, Australia.** Marginal tests indicate the relationship between environmental variables and assemblages individually. The sequential test indicates the relationship between environmental variables and assemblages determined by stepwise multiple regression. Partial R2 values indicate the relationship of a variable once those listed above it have already been fitted to the stepwise model. Bold indicates p < 0.05.

|  |  | Marginal test |  | Sequential test |  |  |
| --- | --- | --- | --- | --- | --- | --- |
|  | Variable | Individual R^2^ | P | Partial R^2^ | Cumulative R^2^ | P |
| Bacterial 16S DNA | Electrical conductivity (μS/cm) | **0.174** | **0.001** | **0.174** | **0.174** | **0.001** |
|  | pH | **0.170** | **0.001** | **0.171** | **0.345** | **0.001** |
|  | Soil Moisture (%) | **0.118** | **0.004** | 0.035 | 0.381 | 0.320 |
|  | Ammonium (mg/Kg) | **0.125** | **0.002** | 0.026 | 0.407 | 0.676 |
|  |  |  |  |  |  |  |
| Bacterial 16S RNA | Soil Moisture (%) | **0.085** | **0.010** | **0.085** | **0.085** | **0.012** |
|  | Electrical conductivity (μS/cm) | **0.077** | **0.025** | 0.063 | 0.148 | 0.080 |
|  | pH | 0.044 | 0.373 | 0.039 | 0.187 | 0.510 |
|  | Ammonium (mg/Kg) | 0.067 | 0.063 | 0.042 | 0.228 | 0.407 |
|  |  |  |  |  |  |  |
| Archaea 16S DNA | Ammonium (mg/Kg) | 0.110 | 0.078 | 0.110 | 0.110 | 0.073 |
|  | pH | 0.105 | 0.100 | 0.058 | 0.168 | 0.319 |
|  | Electrical conductivity (μS/cm) | 0.027 | 0.819 | 0.035 | 0.202 | 0.646 |
|  | Soil Moisture (%) | 0.033 | 0.724 | 0.015 | 0.217 | 0.917 |
