# Supplementary material for: The impact of urbanisation on community structure, gene abundance and transcription rates of microbes in upland swamps of Eastern Australia
Source: PLoS One. 2019 Mar 4;14(3):e0213275. doi: 10.1371/journal.pone.0213275 (PMC6398846; doi:10.1371/journal.pone.0213275)
Supplement: S2 Table — Number of samples out of a total 3 replicates that had sufficient amplification for analysis of DNA and RNA transcribing community analysis (T-RFLP). (DOCX) [file pone.0213275.s002.docx]

**S2 Table. Summary of T-RFLP amplification.** Number of samples out of a total 3 replicates that had sufficient amplification for analysis of DNA and RNA transcribing community analysis (T-RFLP).

| Catchment type | Swamp | Sample depth | T-RFLP Amplification (n/3) | | | |
| --- | --- | --- | --- | --- | --- | --- |
|  |  |  | RNA | | DNA | |
|  |  |  | Bacteria | Archaea | Bacteria | Archaea |
| Intact | Fortress Creek | 0 | 3 | 0 | 3 | 0 |
|  |  | 50 | 3 | 0 | 3 | 2 |
|  | Grand Canyon | 0 | 3 | 1 | 3 | 1 |
|  |  | 50 | 2 | 2 | 3 | 2 |
|  | Michael Eade's Reserve | 0 | 3 | 0 | 3 | 0 |
|  |  | 50 | 3 | 0 | 3 | 2 |
|  | Mt Hay Creek | 0 | 3 | 0 | 3 | 1 |
|  |  | 50 | 3 | 0 | 3 | 3 |
|  | Timmy's | 0 | 3 | 0 | 3 | 0 |
|  |  | 50 | 3 | 0 | 3 | 1 |
|  | Vista | 0 | 3 | 0 | 3 | 0 |
|  |  | 50 | 3 | 0 | 3 | 0 |
| Urbanised | Fifth Avenue | 0 | 3 | 1 | 3 | 1 |
|  |  | 50 | 3 | 1 | 3 | 1 |
|  | Katoomba Falls Rd | 0 | 3 | 0 | 3 | 1 |
|  |  | 50 | 3 | 0 | 3 | 2 |
|  | Marmion Rd | 0 | 3 | 1 | 3 | 1 |
|  |  | 50 | 3 | 0 | 3 | 1 |
|  | Pitt Park | 0 | 3 | 2 | 3 | 2 |
|  |  | 50 | 2* | 1* | 3 | 2* |
|  | Popes Glen | 0 | 3 | 2 | 3 | 3 |
|  |  | 50 | 2* | 1* | 3 | 3 |
|  | Wentworth Falls Lake | 0 | 3 | 0 | 3 | 1 |
|  |  | 50 | 3 | 0 | 3 | 2 |

* Indicates a sample replicate was lost, so these are out of a total of 2 possible replicates rather than 3.
